# Three doses of COVID-19 mRNA vaccine induce class-switched antibody responses in inflammatory arthritis patients on immunomodulatory therapies

**DOI:** 10.3389/fimmu.2023.1266370

**Published:** 2023-10-24

**Authors:** Jenny M. Lee, Alexis Figueroa, Jaiprasath Sachithanandham, Maggie Li, Caoilfhionn M. Connolly, Janna R. Shapiro, Yiqun Chen, Michelle Jones, Venkata Gayatri Dhara, Marilyn Towns, John S. Lee, Stephanie R. Peralta, Aaron M. Milstone, Michael Betenbaugh, Amanda K. Debes, Joel Blankson, Ioannis Sitaras, Steve Yoon, Elizabeth A. Thompson, Clifton O. Bingham, Sabra L. Klein, Andrew Pekosz, Justin R. Bailey

**Affiliations:** ^1^ W. Harry Feinstone Department of Molecular Microbiology and Immunology, Johns Hopkins University Bloomberg School of Public Health, Baltimore, MD, United States; ^2^ Division of Infectious Diseases, Department of Medicine, Johns Hopkins University School of Medicine, Baltimore, MD, United States; ^3^ Division of Rheumatology, Department of Medicine, Johns Hopkins University School of Medicine, Baltimore, MD, United States; ^4^ Department of Chemical and Biomolecular Engineering, Johns Hopkins University, Baltimore, MD, United States; ^5^ Division of Infectious Diseases, Department of Pediatrics, Johns Hopkins University School of Medicine, Baltimore, MD, United States; ^6^ Bloomberg School of Public Health, Johns Hopkins University School of Medicine, Baltimore, MD, United States

**Keywords:** SARS-CoV-2, mRNA vaccines, immunosuppression, inflammatory arthritis, variants of concern, serological response, antibodies, B cells

## Abstract

Patients with inflammatory arthritis (IA) are at increased risk of severe COVID-19 due to medication-induced immunosuppression that impairs host defenses. The aim of this study was to assess antibody and B cell responses to COVID-19 mRNA vaccination in IA patients receiving immunomodulatory therapies. Adults with IA were enrolled through the Johns Hopkins Arthritis Center and compared with healthy controls (HC). Paired plasma and peripheral blood mononuclear cell (PBMC) samples were collected prior to and 30 days or 6 months following the first two doses of mRNA vaccines (D2; HC=77 and IA=31 patients), or 30 days following a third dose of mRNA vaccines (D3; HC=11 and IA=96 patients). Neutralizing antibody titers, total binding antibody titers, and B cell responses to vaccine and Omicron variants were analyzed. Anti-Spike (S) IgG and S-specific B cells developed appropriately in most IA patients following D3, with reduced responses to Omicron variants, and negligible effects of medication type or drug withholding. Neutralizing antibody responses were lower compared to healthy controls after both D2 and D3, with a small number of individuals demonstrating persistently undetectable neutralizing antibody levels. Most IA patients respond as well to mRNA COVID-19 vaccines as immunocompetent individuals by the third dose, with no evidence of improved responses following medication withholding. These data suggest that IA-associated immune impairment may not hinder immunity to COVID-19 mRNA vaccines in most individuals.

## Introduction

Immunocompromised patients are at increased risk of severe COVID-19 (1, 2), mediated by baseline immune dysfunction and impaired host defenses. Patients with rheumatic diseases have increased rates of COVID-19 mortality (3), illustrating the need for effective and optimized vaccines that prevent COVID-19 diseases in this population. While initial SARS-CoV-2 vaccination (either two-dose mRNA or single-dose adenoviral vector dose) elicits high immunogenicity (4–6) and confers protection against severe COVID-19 outcomes among immunocompetent populations (7), a blunted antibody response and an impairment of B- and T-cell responses to SARS-CoV-2 mRNA vaccination has been observed in patients with rheumatic diseases (8–11). Recent publications also suggest that breakthrough COVID-19 disease is more common among fully vaccinated patients with rheumatic diseases than healthy people (1, 12, 13). Extensive research has revealed that immunosuppressive therapies, including temporary suspension of treatment, have a significant impact on vaccine-induced responses in rheumatoid arthritis patients. Specifically, immunosuppressive drugs like methotrexate (MTX) negatively affect both humoral and cellular immune responses to COVID-19 mRNA vaccines, while the temporary withdrawal of MTX has shown to enhance antibody responses (11, 14–17). A systematic evaluation of whether the initial vaccine doses (D2) and third vaccine booster dose (D3) confer protective immune responses against variants of concern (VOCs) that exhibit immune escape during the vaccination periods in the United States remains lacking. Further research is still needed to thoroughly study the impact of immunosuppressive drugs and temporary drug withdrawal on humoral responses against VOCs in IA patients.

During COVID-19 vaccination, the Delta (B.1.617.2) variant (May 2021) and Omicron (B.1.1.529) subvariant BA.1 (Nov,2021) circulated after receipt of the initial two-dose regimen of mRNA vaccines and Omicron subvariants BA.1 and BA.5 (July 2022) variants circulated after receipt of the recommended third booster dose of mRNA vaccines (18–20). Vaccination elicits neutralizing antibodies against the Spike (S) protein, that correlate with protection against SARS-CoV-2 by reducing the rate of severe morbidity and mortality (21–23). Durable memory B cells also represent an important source of protection versus severe SARS-CoV-2 infection (24). Among immunocompetent populations, SARS-CoV-2 vaccines induce robust memory B cell responses and production of neutralizing antibodies upon antigenic re-exposure (25, 26). Little is known about cross-variant neutralizing antibody responses or S-specific B cell maturation in immunocompromised patients, including those with rheumatic diseases. Recent publications have shown that patients treated with lymphocyte-depleting agents, such as rituximab, as well as lymphocyte proliferation inhibitors, such as mycophenolate (including mycophenolate mofetil and mycophenolic acid), exhibit suboptimal antibody responses following two-dose COVID-19 vaccines (8, 9, 21). It is important to inform risk and timing of additional vaccine doses to optimize protection against SARS-CoV-2 infection for patients with rheumatic diseases.

The primary purpose of this study was to assess the immunological response to COVID-19 vaccination among patients with inflammatory arthritis receiving immunomodulatory therapies and to assess cross-reactivity to SARS-CoV-2 variants. The secondary objective was to characterize the impact of initial vaccination on baseline inflammatory arthritis disease status. We hypothesized that increased doses of COVID-19 mRNA vaccines would improve the SARS-CoV-2-specific serological and B cell responses in patients with rheumatic diseases. SARS-CoV-2-specific IgG and IgG subclasses, neutralizing antibody activity, and class-switched B cell frequencies against the ancestral SARS-CoV-2 lineage and VOCs before and after initial doses and a third dose of COVID-19 vaccine were measured and compared to immunocompetent healthy controls (HCs).

## Materials and methods

### Study design and population

This work employed samples from two patient cohorts at the Johns Hopkins Arthritis Center: (1) COVID-19 immunocompromised rhematic patients (COVID-CRP) study (IA Cohort 1) and (2) COVID-19 immunocompromised rheumatic patients (COVID-CRP) booster study (IA Cohort 2), as well as two healthy control (HC) cohorts. IA Cohort 2 received a third dose of homologous COVID-19 mRNA vaccine (Pfizer-BioNTech BNT162b2 or Moderna mRNA-1273) against SARS-CoV-2.

(1) COVID-CRP Study (IA Cohort 1).

For IA Cohort 1, 31 adult patients with a physician verified diagnosis of inflammatory arthritis were enrolled through the Johns Hopkins Arthritis Center (**Table 1**). Enrollments were performed between March 2021 to July 2021. Responses were compared with 77 healthcare workers controls (HC) that received homologous mRNA vaccines as the healthy immunocompetent control population (**Table 1**).

**Table 1:** Baseline characteristics, vaccine platform and immunosuppressive regimen of inflammatory arthritis patients (IA) and healthy controls (HC).

	IA Cohort 1 (pre-D1 and post-D2)	HC Cohort 1 (pre-D1 and post-D2)		IA Cohort 2 (pre- and post-D3)	HC Cohort 2 (pre- and post-D3)	
	Total (N=31)	Total (N=77)		Total (N=97)	Total (N=11)	
Demographics			p-value†			p-value†
**Age, no.(%)**			<0.001			<0.001
median (IQR)	53 (48, 57)	41 (35, 52)		62 (53,70)	-	
18-49	10 (32)	51 (66)		15 (15)	8 (73)	
50-64	20 (65)	17 (22)		44 (45)	3 (27)	
≥65	1 (3)	9 (12)		38 (39)	0 (0)	
**Sex, no.(%)**			0.931			<0.001
Female	20 (65)	49 (64)		76 (78)	4 (36)	
Male	11 (35)	28 (36)		20 (21)	7 (64)	
**Race, no.(%)**			0.581			-
White	23(74)	53 (69)		75 (77)	-	
Non-White	8 (26)	24 (31)		22 (23)	-	
COVID-19 and vaccination history						
Prior SARS-CoV-2 infection*, no.(%)	4 (13)	-		10 (10)	-	
**Days between 2nd and 3rd dose, median (IQR)**	-	-		177 (154,204)	-	
**Vaccine manufacture, no.(%)**			0.011			-
Pfizer-BioNTech (BNT162b2)	21 (68)	68 (88)		61 (62)	-	
Moderna (mRNA-1273)	10 (32)	9 (12)		36 (38)	-	
Disease characteristics						
Inflammatory arthritis diagnosis, no.(%)						
Rheumatoid arthritis	20 (65)			73 (75)		
Psoriatic arthritis	11 (35)			20 (21)		
Others**	-			4 (4)		
Immunosuppression						
Cohort 1 medication groups, no.(%)***						
MTX monotherapy	10 (32)			-		
TNFi monotherapy	12 (39)			-		
Combination MTX and TNFi	5 (16)			-		
Others****	4(13)			-		
Cohort 2 medication groups, no.(%)						
CoStim	-			8 (8)		
Cytokine(i)	-			40 (41)		
JAK(i)	-			17 (18)		
CS DMARD	-			28 (29)		
**With-held therapy for vaccination, no. (%)**	15 (50)			60 (70)		

*By positive prior molecular testing or reactive anti-nucleocapsid antibody at enrollment.

** One patient was diagnosed with myositis, two with scleroderma, one non-applicable.

***Medication groups stratified by rhematologists according to DMARD and biologic medications monotherapy or combination therapy.

**** One patient prescribed tofacitinib, one prescribed secukinumab, two prescribed abatacept.

† Categorical variables (age category, gender, race, etc) were compared between cohorts and controls by chi-square tests.

IQR, interquartile range; MTX, methotraxate; TNF(i), tumor necrosis factor inhibitor; CoStim, T cell costimulation blockers; Cytokine(i), cytokine inhibitors; CS DMARD, conventional synthetic disease modifying antirheumatic drugs; JAK(i), janus kinase inhibitors.

(2) COVID-CRP Booster Study (IA Cohort 2).

For IA Cohort 2, 97 adult patients were enrolled through the Johns Hopkins Arthritis Center. Serum samples were collected immediately before and 30-days following the third dose COVID-19 mRNA vaccines (D3) (**Table 1**). Enrollments were performed from August 2021 to January 2022.

Responses were compared with 11 healthcare workers controls (HC) that also received a third dose of mRNA vaccine as the healthy immunocompetent control population (**Table 1**). IA Cohort 1, IA Cohort 2, and HC studies were approved by the Johns Hopkins University Institutional Review Board, and the participants provided informed written consent prior to enrollment.

### Enzyme-linked immunosorbent assays (ELISAs)

Standardized and validated indirect ELISAs were used to measure the titer of S- and nucleocapsid (N)-specific IgG against the vaccine strain, Delta, and Omicron variants of SARS-CoV-2, as previously described (27–30). Recombinant SARS-CoV-2 S (2 μg/mL) or N proteins (1 μg/mL) engineered at Johns Hopkins or obtained through NCI Serological Sciences Network for COVID-19 were used to coat 96-well ELISA plates (Immulon 4HBX, Thermo Fisher Scientific) and were incubated overnight at 4°C. Plates were washed with phosphate- buffered saline with tween 20 (PBST) wash buffer (Thermo Fisher Scientific), blocked with 3% nonfat milk solution, and incubated for one hour at room temperature (RT). After incubation, the blocking buffer was discarded. Three-fold serially diluted serum samples, monoclonal antibody against the SARS- CoV-2 S protein (positive control; catalog 40150-D001, Sino Biological), and negative control samples were added and incubated for two hours at RT. Plates were washed with PBST, and horseradish peroxidase (HRP)- conjugated secondary IgG antibody (catalog A18823, Invitrogen, Thermo Fisher Scientific) was added at a 1:5,000 dilution and incubated for one hour at RT. For characterization of subclass-specific IgG, we used secondary IgG1 antibody (catalog 9054-05, SouthernBiotech) at a 1:4,000 dilution, IgG2 antibody (catalog 9060-05, SouthernBiotech) at a 1:4,000 dilution, IgG3 antibody (catalog 9210-05, SouthernBiotech) at a 1:4,000 dilution, and IgG4 antibody (catalog 9200-05, SouthernBiotech) at a 1:8,000 dilution. Plates were washed with PBST, and reactions were developed by adding SIGMAFAST OPD (o-phenylenediamine dihydrochloride) solution (MilliporeSigma), followed by a ten-minute incubation at RT. Reactions were stopped by adding 3M hydrochloric acid solution (Thermo Fisher Scientific). The optical density (OD) of each plate was read at 490 nm wavelength on a SpectraMax i3 ELISA Plate Reader (BioTek Instruments). Results were expressed as the log10-transformed area under the curve (AUC) generated from the background- subtracted OD values of the ten three-fold serial dilutions, as previously described (29). A cutoff of 1:180 (or limit of detection [LOD]) was determined for positivity in all ELISA data using prepandemic samples. On the AUC scale, this cutoff (or LOD) was established by calculating the average AUC values of samples with a titer of 1:180.

### Cells and viruses

Vero-E6-TMPRSS2 cells were obtained from the cell repository of the National Institute of Infectious Diseases, Japan and were grown in complete media (CM) consisting of DMEM containing 10% FBS (Gibco, Thermo Fisher Scientific), 1 mM glutamine (Invitrogen, Thermo Fisher Scientific), 1 mM sodium pyruvate (Invitrogen, Thermo Fisher Scientific), 100 U/mL penicillin (Invitrogen, Thermo Fisher Scientific), and 100 μg/mL streptomycin (Invitrogen, Thermo Fisher Scientific). Cells were incubated at 37°C in a humidified incubator with 5% CO2.

SARS-CoV-2 isolates B.1 (hCoV-19/USA/DC-HP00007/2020; B.1, GISAID EPI_ISL_434688), Omicron BA.1 variant (SCV2/USA/MD-HP20874/2021; B.1.1.529, GISAID EPI_ISL_7160424), and Omicron BA.5 (hCoV-19/USA/MD-HP32103-PIDCNSQVGY/2022 GISAID EPI_ISL_15013106) were isolated from Vero-E6-TMPRSS2 cells plated in 24-well plates and grown to 75% confluence (31). The CM was removed and replaced with 150 μL of infection media (IM). IM is identical to CM except with an FBS concentration of 2.5%. 150 μL of the virus transport media containing a SARS-CoV-2 positive clinical swab was added to the culture. The cultures were incubated at 37°C for 2 hours, the inocula were aspirated and replaced with 500 μL of IM, and the cells were incubated at 37°C for up to 5 days. When a cytopathic effect (CPE) was visible in most of the cells, the IM was collected and stored at –65°C. The presence of SARS-CoV-2 was verified by extracting RNA from the harvested supernatant using the QIAGEN QIAamp Viral RNA extraction kit and viral RNA was detected using quantitative rt-PCR. The consensus sequence of the virus isolate did not differ from the sequence derived from the clinical specimen. The infectious virus titer was determined on Vero-TMPRSS2 cells using a 50% tissue culture infectious dose (TCID50) assay (32). Serial 10-fold dilutions of the virus stock were made in IM, and 100 μL of each dilution was then added to the cells in a 96-well plate in hexaplicate. The cells were incubated at 37°C for 4-5 days, fixed with 4% formaldehyde for at least 4 hours and stained with naphthol blue-black overnight. Plates were scored visually for CPE. The TCID50 per mL was determined using the Reed and Muench calculation.

### Microneutralization assay

Plasma nAbs were determined as described for SARS-CoV and modified for SARS-CoV-2 (30, 33). Two- fold dilutions of plasma, starting at a 1:20 dilution, were made using IM. Infectious virus was added to the plasma dilutions at a final concentration of 1 × 10^3 TCID50/mL or 100 TCID50 per 100 μL. The plasma-virus solution was incubated at room temperature for 1 hour, and 100 μL of each dilution was added to 1 well of a 96-well plate of VeroE6-TMPRSS2 cells in hextuplicate. The cells were incubated for 6 hours at 37°C with 5% CO2. The inocula were then replaced with fresh IM, and the cells were incubated at 37°C with 5% CO2 for 2-3 days until CPE is evident in the negative controls. The cells were fixed by the addition of 100 μL of 4% formaldehyde for at least 4 hours at room temperature, and then stained with naphthol blue-black overnight. The nAb titer was calculated as the highest serum dilution that eliminated the CPE in 50% of the wells (NT50) and the AUC was calculated using GraphPad Prism.

### Cell staining and flow cytometry

Peripheral blood mononuclear cells (PBMCs) from inflammatory arthritis patients and healthy controls were used for measuring S-specific B cell responses. Briefly, PBMCs were thawed and washed once in PBS and immediately stained for viability with Invitrogen Live/Dead™ Aqua viability dye and BD Fc Block™ for 10 minutes at room temperature. Cell surface staining was performed in 50uL of 20% BD Horizon™ Brilliant Stain Buffer + PBS with the following surface stain antibody cocktail (clone:flourophore) for 20 minutes at room temperature: CD3/CD14 (UCHT1 and MΦP9:BV510), CD19 (H1B19:APC-H7), CD20 (TH7:BV786), CD10 (HI10a:PE-Cy5), CD27 (M-T271:PE-CF594), CD24 (ML5:BUV395), CD38 (HIT2:BUV661), IgM (MHM88:AF700), IgD (IA6-2:BB790), and spike tetramer probes (BV650, FITC, and PE). Biotinylated spike tetramer probes for ancestral and omicron variants were purchased from R&D Systems and were prepared by incubating the spike protein with streptavidin conjugated to fluorophore at a molar ratio of 4:1 (biotinylated spike protein to streptavidin fluorophore) overnight. The true positive cutoff for S-specific frequency was established by taking the mean ancestral variant S-specific frequency plus 2 standard deviations from three SARS-COV-2 uninfected healthy controls before COVID-19 vaccination. Samples were run on a 4-laser Cytek Aurora spectral flow cytometer and FCS files were analyzed in Flowjo v10 (10.8.1) software.

### Statistical analysis

**S**tatistical calculations were performed using GraphPad Prism 8 (GraphPad Software) and Stata 15 (StataCorp). Data are shown as mean± 95% confidence interval (CI) except where otherwise indicated. Demographics and clinical characteristics were evaluated with descriptive statistics. Comparison between antibody and B cell responses pre- and post-vaccination was performed using the paired t-test. Comparison of antibody and B cell responses among SARS-CoV-2 variants of concern were analyzed using the one-way repeated measures ANOVA test. For associates between clinical parameters and responses, the one-way ANOVA test was used to compare categorical variables and the unpaired t-test to compare binary variables. A P value less than 0.05 was considered significant.

## Results

### Inflammatory arthritis (IA) cohort 1

#### Baseline characteristics

A total of 31 IA patients were included; primarily female (19/31; 61.3%) and white ethnicity (23/31; 74.2%) with median age (IQR) of 53 (48, 57.25) (**Table 1**). Study samples were collected at pre-dose 1 (D1) and 30 days post-dose 2 (D2) between March 10, 2021, and July 16, 2021. Most had a diagnosis of rheumatoid arthritis (20/31; 64.5%) while the remainder had a diagnosis of psoriatic arthritis (11/31; 35.4%). Tumor necrosis factor inhibitor (TNFi) monotherapy was the most common immunosuppressive regimen (12/31; 38.7%) followed by methotrexate (MTX) monotherapy (10/31; 32.2%). Patients received two-dose mRNA-1273 10 (32.3%) or BNT162b2 21 (67.7%). Less than half (14/31 [45.2%]) reported withholding immunomodulatory therapy in the peri-vaccination period.

#### IA patients mount vaccine-induced SARS-CoV-2 S-specific binding antibody and neutralizing antibody responses after the second dose of mRNA COVID-19 vaccine

**Figure 1:**
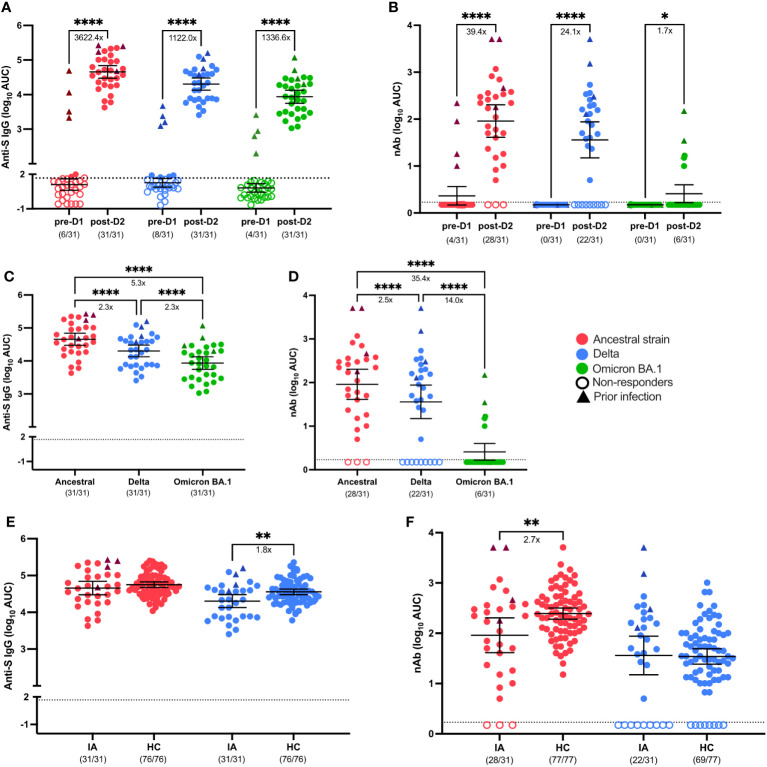
Serological response to SARS-CoV-2 VOCs in Cohort 1 IA patients in comparison of healthy controls (HC). Total SARS-CoV-2 Spike (S)-specific IgG **(A)** and neutralizing antibody (nAb) **(B)** against the ancestral (red), Delta (blue), and Omicron BA.1 (green) variants were measured in IA patients (n=31) prior to dose 1 (pre-D1) and 30 days post-dose 2 (post-D2). Total anti-S IgG **(C)** and nAb levels **(D)** at post-D2 are compared between the ancestral, Delta and Omicron BA.1 variants. Total anti-S IgG **(E)** and nAbs **(F)** against the ancestral and Delta variants are compared between IA patients and HCs (n=77) at post-D2. Dotted lines indicate the limit of detection (LOD), which is 1.67 for anti-S IgG ELISA assay **(A, C, E)** and 0.23 for the nAb assay **(B, D, F)**. Open circles represent non-responders with negative serological responses that fall below the LOD value. Triangles represent patients with known prior SARS-CoV-2 infection. The mean± 95% CI are shown in each panel. Significance is tested using paired t test **(A, B)**, one- way repeated measures ANOVA **(C, D)**, and unpaired t-tests **(E, F)**. *p < 0.05, **p < 0.01, ***p < 0.001, and ****p <0.0001. Fold changes (x) are labeled below the significance lines. Number of positive samples out of the total number of samples tested are indicated in parentheses.

The magnitude and breadth of the pre-dose 1 (D1) and post-dose 2 (D2) IgG responses to SARS-CoV-2 S protein were assessed in plasma samples collected from Cohort 1 IA patients from March 2021 to July 2021, which corresponded with the Delta and early Omicron COVID-19 waves (19). IgG binding to the S protein of the ancestral lineage (3622.4-fold increase over pre-D1; p< 0.0001), S protein from the Delta variant (1122.0-fold increase over pre-D1; p< 0.0001), and the S protein from the Omicron BA.1 variant (1336.6-fold increase over pre-D1; p< 0.0001) were significantly elevated following D2 of the COVID-19 mRNA vaccines (**Figure 1A**). Likewise, the mean neutralizing antibody (nAb) responses against the ancestral lineage (39.4-fold increase; p< 0.0001), the Delta variant (24.1-fold increase; p< 0.0001), and the Omicron BA.1 variant (1.7-fold increase; p< 0.05) also increased significantly after D2 as compared with pre-vaccination (**Figure 1B**). Although vaccination induced antibody responses that recognized the S protein from all of the lineages of SARS-CoV-2 tested, the responses to the Omicron BA.1 variant (5.3-fold decrease in anti-S IgG; 35.4-fold decrease in nAb; p< 0.0001) and Delta variant (2.3-fold decrease in anti-S IgG; 2.5-fold decrease in nAb; p< 0.0001) were significantly lower when compared to the ancestral lineage after D2 (**Figures 1C, D**). While all individuals seroconverted as judged by S protein binding antibodies, the number of responders was lower for neutralizing antibodies, with seroconversion occurring in 28/31 individuals for vaccine lineage, 22/31 for Delta variant and 6/31 for Omicron BA.1 variant. Four individuals with known COVID-19 infections had higher anti-S antibody titers and detectable neutralizing antibody levels to the ancestral strain at the time of vaccination. As expected, these individuals had the highest and broadest boost in antibody reactivity after vaccination. We next compared post-vaccination serological responses between IA patients and vaccinated HCs, which were used as immunocompetent controls, to determine if there were differences in vaccine-induced immunity between immunocompromised IA patients and immunocompetent individuals. After receipt of two mRNA doses, anti-ancestral S IgG responses were similar among IA and HC, but antibody responses against Delta-S were lower in IA than in HC (1.8-fold decrease; p< 0.01; **Figure 1E**). In contrast, the mean post-D2 nAb response against the ancestral virus were significantly lower in IA compared to HC (2.7-fold decrease; p< 0.01), but the IA and HC groups had similar nAb responses against the Delta variant (**Figure 1F**). A similar proportion of the HC had detectable neutralizing antibodies to both the vaccine lineage (28/31 versus 80/80) and the Delta variant (22/31 versus 70/79) compared to the IA cohort. As expected, individuals with a prior SARS-CoV-2 infection tended to have stronger antibody responses (triangles). To determine the effect of different immunomodulatory regimens on vaccine-induced immunity in IA patients, post-D2 serological responses were stratified by immunomodulatory regimen (i.e., methotrexate monotherapy [MTX], anti-tumor necrosis factor inhibitor monotherapy [TNFi] or combination MTX+TNFi) and by presence/absence of peri-vaccination withholding of medications. There were no statistically significant differences in anti-S IgG responses (**Supplementary Figures 1A–C, G-I**) or nAb responses (**Supplementary Figures 1D–F, J–L**) by either immunosuppressive regimen or peri-vaccination withholding of therapy. We also evaluated the intersection of sex and aging on SARS-CoV-2 vaccine-induced antibody responses against VOCs in IA patients, and the data were disaggregated by sex and age at post-D2. There were no statistically significant differences in either anti-S IgG responses (**Supplementary Figure 2A**) or nAb responses (**Supplementary Figure 2B**) against the ancestral strain, Delta variant, or Omicron BA.1 variant. We also did not observe statistically significant differences in either anti-S IgG responses (**Supplementary Figure 2C**) or nAb responses (**Supplementary Figure 2D**) between younger (18-49) and older (50-64) aged individuals, regardless of sex. Taken together, IA patients exhibited lower IgG responses to the Delta variant and reduced nAb responses to the ancestral virus compared to HC, with no significant differences observed based on different immunomodulatory regimens or medication withholding.

#### IA patients mount impaired S-specific B cell responses after the second dose of mRNA COVID-19 vaccine

**Figure 2:**
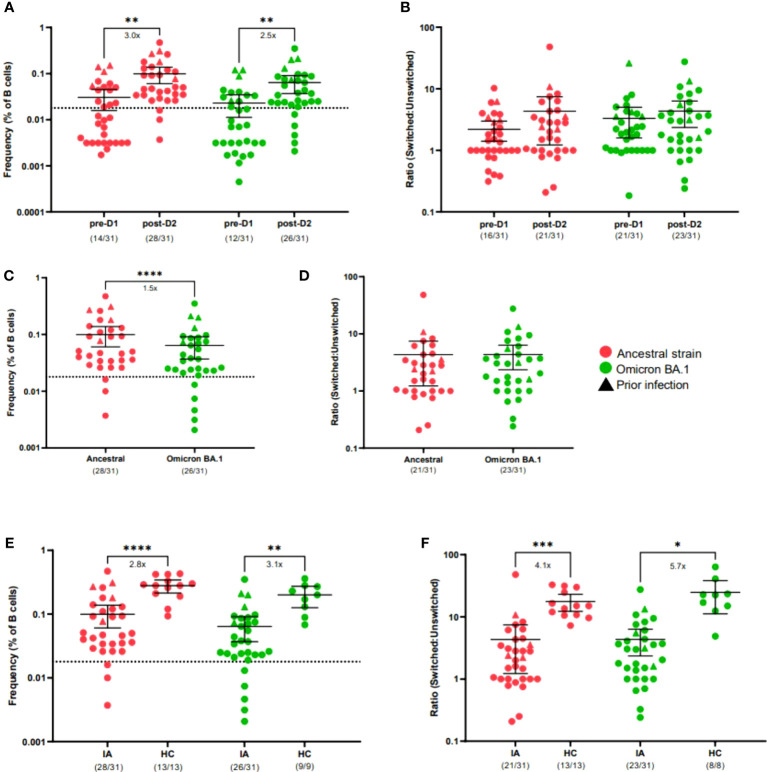
B cell response to SARS-CoV-2 VOCs in Cohort 1 IA patients in comparison of healthy controls (HC). Total SARS-CoV-2 S-specific B cells **(A)** and ratio of switched to unswitched S-specific B cells **(B)** reactive against ancestral (red) or cross-reactive against Omicron BA.1 (green) variants were measured in IA patients (n=31) prior to dose 2 (pre-D1) and 30 days post-dose 2 (post-D2). Total S-specific B cells **(C)** and ratios **(D)** at post-D2 are compared between B cells reactive against ancestral or cross-reactive against Omicron BA.1 variants. Total S-specific B cells **(E)** and ratios **(F)** of B cells reactive against ancestral and cross-reactive against Omicron BA.1 variants are compared between IA patients and HCs (n=11 or n=9) at post-D2. Dotted lines indicate the background S-specific B cell frequency for an unvaccinated control, which is 0.018 **(A, C, E)**. Triangles represent patients with known prior SARS-CoV-2 infection. The mean± 95% CI are shown in each panel. Significance is tested using one-way repeated measures ANOVA **(A, B, E, F)** or unpaired t-tests **(C, D)**. *p < 0.05, **p < 0.01, ***p < 0.001, and ****p <0.0001. Fold changes (x) are labeled below the significance lines. For B cell frequencies, the number of positive samples out of the total number of samples tested are indicated in parentheses. For B cell ratios, the number of samples above a ratio of one out of the total number of samples tested are indicated in parentheses.

To assess the cellular response to COVID-19 vaccines in IA patients, we implemented a spectral flow cytometry-based assay to determine frequencies of SARS-CoV-2 S-specific cells from the B cell compartment of peripheral blood samples from IA patients pre-D1 and post-D2 (**Supplementary Figure 3**). In agreement with anti-S IgG binding titers, total ancestral (p < 0.01) and omicron BA.1 (p < 0.01) S-specific B cell frequencies increased significantly from baseline to 30-days post-D2 (**Figure 2A**). Overall, 90% and 84% of IA patients had ancestral and omicron BA.1 S-specific B cell responses, respectively, above the positive cutoff for the assay. Although most IA patients had detectable omicron BA.1 S-specific B cells, the magnitude of this response was 1.5-fold lower compared to the ancestral S-specific response (p < 0.0001; **Figure 2C**). We also measured the ability of IA patients to produce a fully matured B cell response by calculating the ratio of class-switched (IgM- and IgD-) to unswitched (IgM+ and/or IgD+) S-specific B cells. There was no significant increase in the class-switched S-specific B cell ratio from baseline to 30-days post-D2 (**Figure 2B**) with only 68% of IA patients having a class-switched ratio above 1 for ancestral variant and 74% of IA patients having a class-switched ratio above 1 for omicron BA.1 variant. When comparing these ratios, similar class-switching rates were observed for both the ancestral and omicron BA.1 response in IA patients post-D2 (**Figure 2D**). No significant differences among the immunomodulatory drug regimens were observed on the B cell response in IA patients assayed at this time point (**Supplementary Figures 4A–D**). We also compared B cell frequencies in IA patients to HCs assayed 6-8 months after D2. IA patients had significantly lower frequencies of ancestral (2.8-fold, p < 0.0001) and omicron BA.1 (3.1-fold, p < 0.01) S-specific B cells compared to HCs (**Figure 2E**). Consistent with these findings, IA patients also demonstrated significantly lower class-switching ratios of S-specific responses for both ancestral (4.1-fold, p < 0.001; **Figure 2F**) and omicron BA.1 variants (5.7-fold, p < 0.05; **Figure 2F**). Collectively, these data indicate that most IA patients can mount S-specific B cell responses post-D2, but these responses are less frequent and less class-switched compared to HCs.

### Inflammatory arthritis (IA) cohort 2

#### Baseline characteristics

A total of 97 participants were included; primarily female (75/97; 77.3%) and white ethnicity (75/97; 77.3%) with median age (IQR) of 62 (53, 70) (**Table 1**). Study samples were collected at pre-dose 3 (D3) and 30 days post-D3 between August 19, 2021, and January 13, 2022. Most had a diagnosis of rheumatoid arthritis (73/97; 75%) while the remainder had a diagnosis of psoriatic arthritis (20/97; 21%) or others (4/97; 4%). Cytokine inhibitor [Cytokine(i)] monotherapy was the most common immunosuppressive regimen (40/97; 41%) followed by conventional synthetic disease modifying antirheumatic drugs (CS DMARD) therapy (28/97; 29%), Janus kinase inhibitor [JAK(i)] monotherapy (17/97; 18%), and T cell stimulation blocker treatment (CoStim) (8/97; 8%). Patients received a third dose mRNA-1273 (36/97; 38%) or BNT162b2 (61/97; 62%). More than half (60/97; 70%) reported withholding immunomodulatory therapy in the peri-vaccination period.

#### IA patients mount robust and comparable SARS-CoV-2 vaccine-induced serological responses to healthy controls after receipt of a third dose of mRNA COVID-19 vaccine

**Figure 3:**
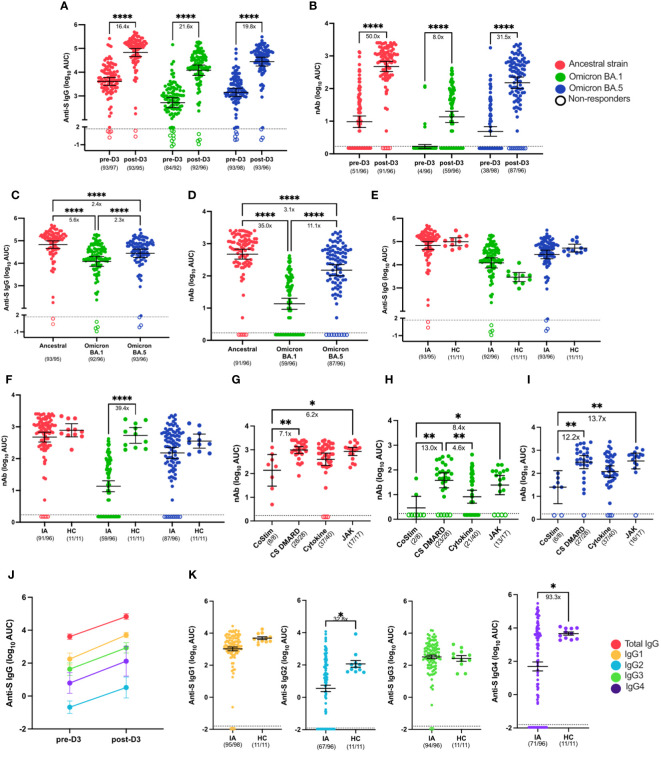
Serological responses to SARS-CoV-2 VOCs in Cohort 2 IA patients in comparison of healthy controls (HC). Total SARS-CoV-2 Spike (S)-specific IgG **(A)** and neutralizing antibody (nAb) **(B)** against the ancestral strain (red), Omicron BA.1 (green), and Omicron BA.5 (blue) variants measured in IA patients (n=97) prior to dose 3 (pre-D3) and 30 days post-dose 3 (post-D3). Total anti-S IgG **(C)** and nAb levels **(D)** at post-D3 were compared between the ancestral strain, Omicron BA.1, and Omicron BA.5 variants. Total anti-S IgG **(E)** and nAbs **(F)** against the ancestral strain, Omicron BA.1, and Omicron BA.5 variants were compared between IA patients and HC (n=11) at post-D3. nAb versus the ancestral strain **(G)**, Omicron BA.1 **(H)**, and Omicron BA.5 **(I)** were measured in IA patients at post-D3 and stratified by immunosuppressive medication received: T cell co-stimulation modulators (CoStim), conventional synthetic disease modifying antirheumatic drugs (CS DMARD), cytokine modulators (Cytokine), and Janus Kinase inhibitors (JAK). SARS-CoV-2 Spike (S)-specific IgG subclasses (IgG1-4): IgG1 (yellow), IgG2 (blue), IgG3 (green), and IgG4 (purple) against the ancestral strain are presented and connected by lines to show the changes of serological responses in IgG subclasses from pre-D3 to post-D3 **(J)**. Comparison of anti-S IgG subclass-specific antibody levels against the ancestral strain were made between IA patients and HC at post-D3 **(K)**. Dotted lines indicate the limit of detection (LOD), which is 1.67 for the anti-S IgG ELISA assay **(A, C, E G)**, 0.23 for the nAb assay **(B, D, F, G-I)**, and -1.96 for the subclass-specific IgG ELISA assay **(K)**. Open circles represent non-responders with negative serological responses that fall below the LOD value. The mean± 95% CI are shown in each panel. Significance is tested using paired t test **(A, B)**, one-way repeated measures ANOVA **(C, D)**, unpaired t-tests **(E, F, K)**, and ordinary one-way ANOVA **(G-I)**. *p < 0.05, **p < 0.01, ***p < 0.001, and ****p <0.0001. Fold changes (x) are labeled below the significance lines. Number of positive samples out of the total number of samples tested are indicated in parentheses.

To evaluate the impact of a third booster dose (D3) on vaccine-induced immunity in IA patients, samples were collected from Aug 2021 to Jan 2022, which corresponded with the beginning of the Omicron wave (19). IgG binding to the S protein of the ancestral strain (16.4-fold increase; p< 0.0001), Omicron BA.1 variant (21.6-fold increase; p< 0.0001), and Omicron BA.5 variant (19.8-fold increase; p< 0.0001) increased significantly following D3 as compared with prior to the booster dose (**Figure 3A**). Likewise, nAb responses against the ancestral strain (50.0-fold increase; p< 0.0001), Omicron BA.1 variant (8.0-fold increase; p< 0.0001), and Omicron BA.5 variant (31.5-fold increase; p< 0.0001) increased significantly after D3 as compared with pre-D3 (**Figure 3B**). Although third-dose vaccination induced responses to all strains of SARS-CoV-2, responses to the Omicron BA.1 variant (5.6-fold decrease in anti-S IgG; 35.0 -fold decrease in nAb; p< 0.0001) and Omicron BA.5 variant (2.4-fold decrease in anti-S IgG; 11.1-fold decrease in nAb; p< 0.0001) were significantly lower when compared to responses to the ancestral strain after D3 (**Figures 3C, D**). We next compared post-D3 serological responses between IA patients and vaccinated HCs to determine if there were differences in vaccine-induced immunity between immunosuppressed IA patients and HCs. After receipt of the third mRNA dose, anti-S IgG responses against the ancestral strain, Omicron BA.1, and Omicron BA.5 variants were similar among IA and HCs (**Figure 3E**). Similarly, post-D3 nAb responses against the ancestral virus and Omicron BA.5 were comparable in IA and HCs, but the nAb responses to Omicron BA.1 were significantly lower in IA than HC (39.4-fold decrease; p< 0.0001) (**Figure 3F**). It is important to note that not all IA patients had detectable nAb responses to vaccine (91/96), BA.1 (59/96) or BA.5 (87/96) compared to the HC cohort where all individuals had nAb response after D3. To determine the effect of different immunomodulatory regimens on vaccine-induced immunity in IA patients, post-D3 serological responses were stratified by immunomodulatory regimen (i.e., T cell co-stimulation modulator therapy [CoStim], conventional synthetic disease modifying antirheumatic drug therapy [CS DMARD], cytokine modulator therapy [Cytokine], or Janus Kinase inhibitor therapy [JAK]) and by presence/absence of peri-vaccination withholding of medications. There were no significant differences in the magnitude of the anti-S IgG responses to ancestral strain (**Supplementary Figure 5A–C**) associated with any of the immunosuppressive regimens, but we observed significantly decreased nAb responses in the CoStim immunomodulatory regimen (**Figure 3G**). Similar results were observed in response to SARS-CoV-2 VOCs (**Figures 3H, I**). Peri-vaccination withholding of immunomodulatory therapy did not significantly affect either anti-S IgG responses (**Supplementary Figure 5D–F**) or nAb responses (**Supplementary Figure 5G–I**). To evaluate the intersection of sex and aging on SARS-CoV-2 vaccine-induced antibody responses in IA patients, data were disaggregated by sex and age at post-D3. There were no significant differences in either anti-S IgG responses (**Supplementary Figure 2E**) or nAb responses (**Supplementary Figure 2F**) against the ancestral strain, Omicron BA.1, and Omicron BA.5 variants. We also did not observe significant differences in either anti-S IgG responses (**Supplementary Figure 2G**) or nAb responses (**Supplementary Figure 2H**) across age groups of 18-49, 50-64, and ≥65. Overall, the majority of IA patients had detectable antibody responses that cross reacted with the BA.1 and BA.5 variants, though the nAb responses were somewhat less robust when compared to HC.

Our research further provided an in-depth analysis of IgG subclasses (IgG1-4) profiles to understand the roles of IgG subclasses in response to SRS-CoV-2 infection in IA patients. The abundance of SARS-CoV-2 ancestral S-specific IgG subclasses was assessed in IA patients after D2 and D3 (**Supplementary Figure 6**). All IgG subclasses increased significantly from pre-D1 to post-D2 (**Supplementary Figure 6A**), with IgG1 and IgG3 being predominantly abundant after D2 in IA patients (**Supplementary Figures 6B, C**). Similarly, we found a significant increase of IgG subclasses titers after D3 (**Supplementary Figure 6D**). However, there were differential patterns in the IgG subclass profiles in IA patients between post-D2 (**Supplementary Figure 6C**) and post-D3 (**Figure 3J**). IgG1 and IgG3 were the predominant subclasses after D2, whereas IgG1 remained the most abundant subclass followed by IgG3 and IgG4, and IgG2 after D3 (**Supplementary Figure 6E**). Comparison of IgG subclasses between IA and vaccinated HCs (**Supplementary Figures 6F, G**) revealed differences in the IgG subclass responses following D3 between immunocompromised IA patients and immunocompetent individuals (**Figure 3K**). IA patients mounted similar IgG1 and IgG3 antibody responses to HCs (**Figure 3K**). However, anti-S IgG2 (32.8-fold decrease; p<0.05) and IgG4 (93.3-fold decrease; p<0.05) abundance was significantly lower in IA patients as compared with HC (**Figure 3K**). We also assessed whether switched S-specific B cell frequencies would correlate with total anti-S IgG post-D3 in IA patient samples (**Supplementary Figure 6H**). We found that the frequency of switched S-specific B cells positively correlated with anti-S IgG levels in the sera post-D3 for ancestral S (**Supplementary Figure 6H**). In conclusion, our study showed that SARS-CoV-2 vaccines induced a significantly rise in IgG subclass titers in IA patients, but the relative abundance of these subclasses changed after D3. We also observed a decrease in IgG2 and IgG4 levels in IA patients compared to HC after D3, highlighting notable distinctions in the immune responses between these two populations.

#### IA patients on co-stimulatory molecule inhibitor therapy have less frequent S-specific B cells after the third dose of COVID-19 vaccine

**Figure 4:**
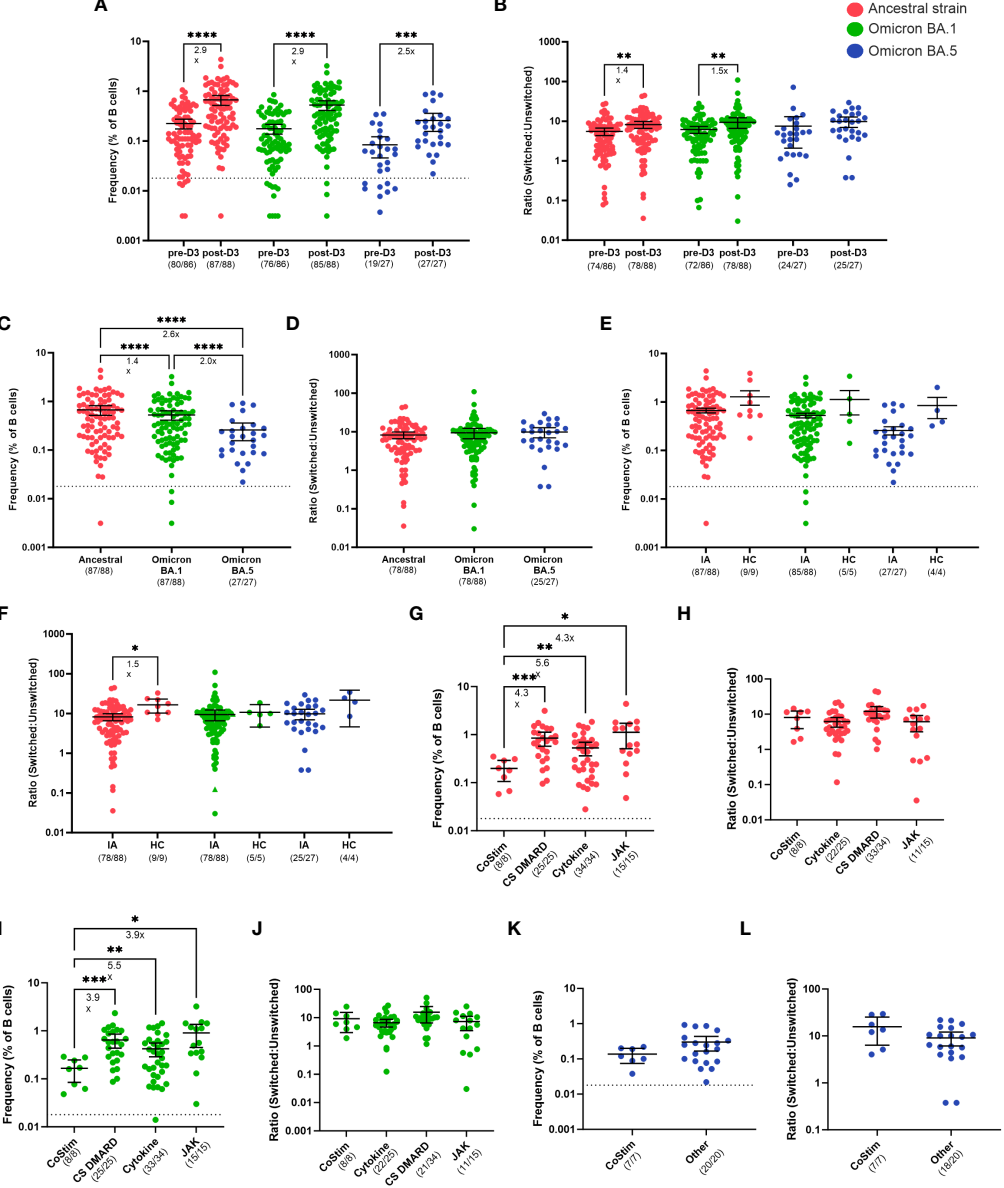
B cell responses to SARS-CoV-2 VOCs in Cohort 2 IA patients in comparison of healthy controls (HC). Total SARS-CoV-2 S-specific B cells **(A)** and ratio of switched to unswitched S-specific B cells **(B)** reactive against ancestral strain (red), cross-reactive against Omicron BA.1 (green), and cross-reactive against Omicron BA.5 (blue) variants measured in IA patients prior to dose 3 (pre-D3, n=86) and 30 days post-dose 3 (post-D3, n=88). Total S-specific B cells **(C)** and ratio **(D)** at post-D3 were compared between B cells reactive against ancestral, cross-reactive against Omicron BA.1, and cross-reactive against Omicron BA.5 variants. Total S-specific B cells **(E)** and ratios **(F)** of B cells reactive against ancestral, cross-reactive against Omicron BA.1, and cross-reactive against Omicron BA.5 variants were compared between IA patients and HC (n=9, 5, and 4, respectively) at post-D3. Total S-specific B cells **(G)** and ratios **(H)** against ancestral variant were measured in IA patients at post-D3 and stratified by immunosuppressive medication received: T cell co-stimulation modulators (CoStim), conventional synthetic disease modifying antirheumatic drugs (CS DMARD), cytokine modulators (Cytokine), and Janus Kinase inhibitors (JAK). Total S-specific B cells **(I)** and ratios **(J)** cross-reactive against Omicron BA.1 variant were measured in IA patients at post-D3 and stratified by immunosuppressive medication received. Total S-specific B cells **(K)** and ratios **(L)** cross-reactive against Omicron BA.5 variant were measured in IA patients at post-D3 and stratified by immunosuppressive medication received: CoStim or all other medications (other). Dotted lines indicate the true positive cutoff for S-specific B cell frequency which is 0.018 **(A, C, E, G, I, K)**. For the analysis of B cell ratios, the dotted lines indicate a ratio of 1 **(B, D, F, J, L)**. The mean± 95% CI are shown in each panel. Significance is tested using one- way repeated measures ANOVA **(A-J)** or unpaired t-tests **(K, L)**. *p < 0.05, **p < 0.01, ***p < 0.001, and ****p <0.0001. Fold changes (x) are labeled below the significance lines. For B cell frequencies, the number of positive samples out of the total number of samples tested are indicated in parentheses. For B cell ratios, the number of samples above a ratio of one out of the total number of samples tested are indicated in parentheses.

We next investigated whether B cell responses could improve in IA patients after a third dose of COVID-19 vaccination. Consistent with anti-S IgG binding titers, total ancestral (2.9-fold, p < 0.0001), omicron BA.1 (2.9-fold, p < 0.0001), and omicron BA.5 (2.5-fold, p < 0.001) S-specific B cell frequencies were significantly increased from D2 to 30-days post-D3 (**Figure 4A**). Overall, 99%, 86%, and 100% of IA patients had detectable ancestral, omicron BA.1, and omicron BA.5 B cell responses, respectively, above the positive cutoff for the assay post-D3. IA patients had significantly greater S-specific class-switching ratios after D3 for ancestral (1.4-fold, p < 0.01) and omicron BA.1 (1.5-fold, p <0.01) variants, but not for the omicron BA.5 variant (**Figure 4B**). The magnitude of the ancestral S-specific response was significantly higher post-D3 (**Figure 4C**) compared to omicron BA.1 (1.4-fold, p < 0.0001) and omicron BA.5 (2.6-fold, p < 0.0001) responses, but no differences were observed in the class-switching ratios between B cells specific for these variants (**Figure 4D**). We also assayed HCs sampled 30-days post D3 and found that IA patients had similar frequencies of S-specific B cells for all variants tested (**Figure 4E**). Furthermore, minimal class-switching defects were observed in IA patients, with 88% of samples having a class-switched ancestral S-specific ratio above 1 (**Figure 4F**); this class-switching ratio, however, was 1.5-fold lower than HCs (p < 0.05). There were significant effects of different immunomodulatory drug regimens on B cell frequencies. IA patients taking co-stimulatory molecule inhibitors (CoStim) had significantly lower frequencies of ancestral S-specific (**Figure 4G**) and omicron BA.1 (**Figure 4I**) S-specific B cells compared to IA patients on any other immunomodulatory therapies. These effects were not observed in omicron BA.5 S-specific B cell frequencies (**Figure 4K**). Furthermore, no defects in class-switching were observed as a result of different drug regimens, indicating that co-stimulatory molecules likely only affect the frequency of S-specific B cells (**Figures 4H, J, L**). These results indicate that most IA patients on immunomodulatory therapies benefit from a third of COVID-19 vaccination, with correction of B cell deficits observed after D2. Only those IA patients on CoStim demonstrated residual impaired B cell responses relative to other IA patients after D3.

## Discussion

In the present study, we performed a comprehensive analysis using IgG ELISA assays, live virus microneutralization assays, and flow cytometry to characterize antibody responses and spike (S)-specific B cell responses in IA patients receiving immunomodulatory therapies before and after the second (D2) or third (D3) dose of SARS-CoV-2 ancestral mRNA vaccines. In previous reports, patients with inflammatory rheumatic (IA) diseases often experience impaired responses to SARS-CoV-2 vaccines under immunosuppressive treatment and are at higher risk of severe COVID-19 (34, 35). In our study, we provide evidence that mRNA vaccines significantly enhance antibody responses against ancestral strain and SARS-CoV-2 VOCs in most IA patients following D2 and D3. Importantly, most IA patients were able to achieve antibody levels comparable to those of HC after D3, effectively mitigating the initial deficit observed in D2 vaccine series. However, there were a number of IA patients with no detectable nAb levels after D2 and D3, indicating some reduction in the ability of a subset of IA patients to respond strongly to COVID-19 vaccination. Our study found no sex or age bias in vaccine-induced serological responses among IA patients.

We also examined the subclass-specific anti-S antibody profile in IA patients. Previous research has shown that IgG antibody responses to viral antigens are mostly composed of IgG1 and IgG3. IgG1 and IgG3 are potent triggers of effector mechanisms because of stronger antibody binding, while IgG2 and IgG4 often induce muted responses that are non-inflammatory (36). Our data also revealed that IgG1 and IgG3 were the most abundant subclasses in IA patients after D2, which resembled the dominant antiviral IgG subclasses reported in literature (36). However, this distinct subclass profile was no longer observed in IA patients after D3, with IgG1 being the most abundant subclass followed by comparable IgG3 and IgG4 levels. This elevated level of IgG4 was observed in both IA and HC after receipt of third dose SARS-CoV-2 vaccines, which was consistent with recently published literature showing class switch toward IgG4 after repeated SARS-CoV-2 mRNA vaccination (37). It is also notable to point out reduced IgG2 and IgG4 levels in IA patients compared to HC after D3, suggesting that non-inflammatory IgG2 and IgG4 might play a distinguishable role in the vaccine-induced immunity of SARS-CoV-2 mRNA vaccines among IA patients.

Moreover, we detected ancestral S-specific B cells in 90% of IA patients at 30-days post-D2 and 99% of IA patients at 30-days post-D3. Most IA patients and HCs were also able to produce S-specific B cells cross-reactive against variants of concern including Omicron BA.1 or BA.5 after D2 or D3 despite being vaccinated only with ancestral S. However, we identified deficits post-D2 in S-specific B cell frequencies and class-switching in IA patients relative to HCs. Notably, a third dose of the COVID-19 vaccine corrected these deficits for most IA patients, with only those on CoStim demonstrating persistently reduced S-specific B cell frequencies and reduced nAb levels post-D3.

Our results align with prior studies of rheumatoid arthritis (RA) patients, which demonstrated increased-S specific antibodies and RBD-specific B cell frequencies after D2 or D3 (38, 39). However, a full analysis of both the magnitude and quality of the antibody responses and S-specific B cell response after D2 and D3 in IA patients on various immunomodulators has been lacking. We observed that IA patients have similar antibody responses and frequencies of S-specific B cells, regardless of immunomodulatory therapy or medication withholding during the D2 vaccination period. This reveals that neither immunosuppressive drugs such as methotrexate (MTX) or tumor necrosis factor inhibitors (TNFi), nor immunosuppressive treatment withdrawal, would affect antibody or B cell responses after IA patients received completed mRNA vaccination (post-D2). These findings contradict the established research that demonstrates the impact of MTX, but not TNFi, on weakening humoral and cellular immune responses to SARS-CoV-2 mRNA and other vaccines, including influenza vaccines (8, 11, 16, 17, 40, 41). Similarly, our results are inconsistent with previous research that suggests immunosuppressive treatment can enhance vaccine responses (14). The lack of significant effect of methotrexate on antibody titers in our study might be the result of lack of power due to fewer participants relative to some prior studies. However, it is interesting that IA patients generated both reduced titers of nAbs and reduced frequencies of class-switched S-specific B cells after D2, indicating that both the magnitude and the quality of their humoral immune response was reduced. This could potentially be explained by lack of T cell help due to immunosuppressive medications. This hypothesis is supported by the observation that the most profound nAb and B cell deficits were observed in patients on CoStim, which blocks CD28 activation of naïve T cells during antigen presentation, which may lead to less T cell help for B cells. Alternatively, immunosuppression may lead to less activation of memory B cells after vaccination. Consistent with this explanation, prior research found that RA patients showed impaired B cell activation 7 days after COVID-19 vaccination (38).

This study has several limitations. The relatively small sample size in each cohort may restrict the generalizability of our findings to a broader IA population. Cell samples were not available for the majority of HCs. The lack of identical D2 and D3 cohorts hinders a longitudinal analysis to assess the efficacy of mRNA vaccines. Additionally, the inclusion of various IA conditions, such as RA and psoriatic arthritis (PsA), introduced disease as a variable, which we did not include in our analyses. The small sample size within each immunomodulatory medication makes it challenging to draw definitive conclusions about the specific effects of these immunomodulatory medications on vaccine-induced responses. Notably, we exclude two patients who received Rituximab (RTX) with significantly reduced responses from the data analysis, potentially underscoring the negative impact of this medication on COVID-19 vaccine responses. It is also notable that several study subjects had positive S-specific B cell responses at the pre-D1 timepoint, despite negative anti-nucleocapsid titers and no documented history of SARS-CoV-2 infection. We speculate that these individuals may have had prior, undocumented SARS-CoV-2 exposures, or that these responses could represent cross-reactivity with SARS-CoV-2 and endemic coronaviruses. Positive anti-S titers were not observed pre-D1 in most individuals with S-specific B cells, possibly because the B cell assay is more sensitive than the antibody assay, or because some S-specific B cells are resting and not secreting antibody. We also did not assess the degree of somatic mutation in S-specific B cells of IA patients compared to HCs, nor the magnitude of the CD4+ S-specific T cell response. Given these limitations, further investigations with larger sample sizes and diverse IA cohorts are necessary to validate our findings and explore the impact of specific IA disease status and medication regimens on SARS-CoV-2 mRNA vaccine-induced responses.

It is worth noting that unlike other moderately to severely immunocompromised populations, such as solid organ transplant recipients and cancer patients, which exhibit significantly lower neutralization activity and cellular responses following a third dose of SARS-CoV-2 mRNA vaccines, our study observed a positive and comparable vaccine outcome in most IA patients compared to healthy individuals (40–42). We have demonstrated that Anti-S IgG, nAb and S-specific B cells develop in most IA patients following D2 and D3, and that D3 is crucial for the IA patient population to develop comparable antibody responses with HCs and normal frequencies of class-switched S-specific B cells. Some patients on CoStim may require more than 3 vaccine doses or vaccination with secession of treatment to achieve a normal nAb and B cell responses. These data have important implications for clinical management of IA patients, and for understanding human vaccine responses in the context of autoimmunity and immune suppression.

## Data availability statement

The raw data supporting the conclusions of this article will be made available by the authors, without undue reservation.

## Ethics statement

The studies involving humans were approved by Institutional Review Board of Johns Hopkins School of Medicine. The studies were conducted in accordance with the local legislation and institutional requirements. The participants provided their written informed consent to participate in this study.

## Author contributions

JML: Conceptualization, Data curation, Formal Analysis, Investigation, Writing – original draft, Writing – review & editing, Visualization. AF: Formal Analysis, Investigation, Methodology, Writing – original draft, Writing – review & editing, Conceptualization, Data curation, Visualization. JS: Formal Analysis, Investigation, Writing – review & editing. ML: Formal Analysis, Investigation, Writing – review & editing. CC: Writing – review & editing, Conceptualization, Writing – original draft. JRS: Writing – review & editing. YC: Writing – review & editing, Resources. MJ: Writing – review & editing, Project administration. VGD: Writing – review & editing, Resources. MT: Writing – review & editing, Project administration. JSL: Writing – review & editing, Methodology. SP: Writing – review & editing. AM: Writing – review & editing, Resources. MB: Writing – review & editing, Resources. AD: Writing – review & editing. JB: Resources, Writing – review & editing. IS: Writing – review & editing. SY: Writing – review & editing. ET: Writing – review & editing, Formal Analysis, Investigation. CB: Writing – review & editing, Conceptualization, Funding acquisition, Methodology, Resources, Supervision, Writing – original draft. SK: Formal Analysis, Investigation, Writing – review & editing, Conceptualization, Methodology, Supervision, Writing – original draft. AP: Methodology, Resources, Supervision, Writing – original draft, Writing – review & editing, Formal Analysis, Investigation. JRB: Conceptualization, Formal Analysis, Investigation, Methodology, Supervision, Writing – original draft, Writing – review & editing, Data curation, Project administration, Resources.
