# Supplementary figures and images for: Three doses of COVID-19 mRNA vaccine induce class-switched antibody responses in inflammatory arthritis patients on immunomodulatory therapies
Source: Front Immunol. 2023 Oct 24;14:1266370. doi: 10.3389/fimmu.2023.1266370 (PMC10646683; doi:10.3389/fimmu.2023.1266370)

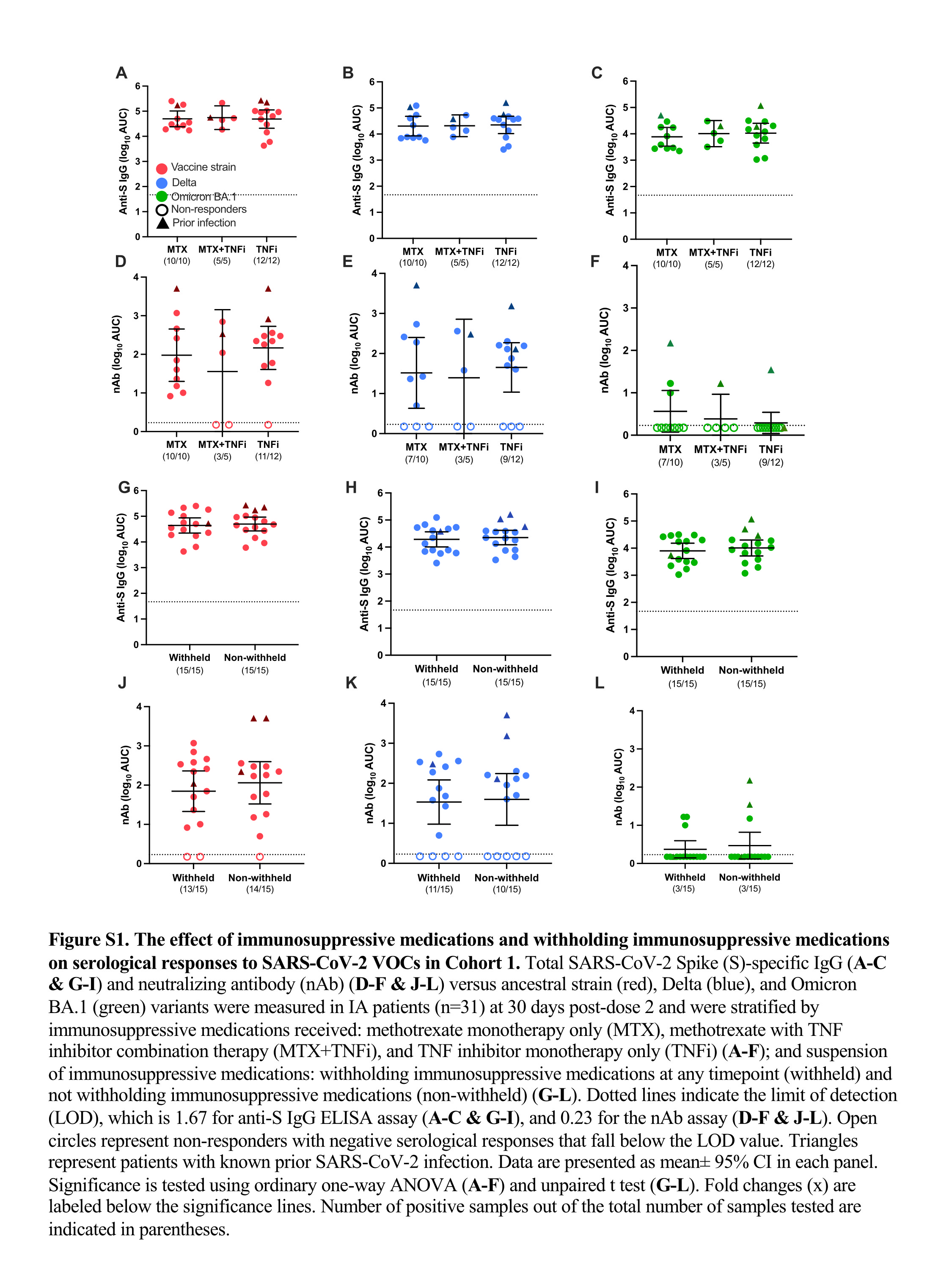

Supplement: Supplementary file 1 [file Image_1.tif]

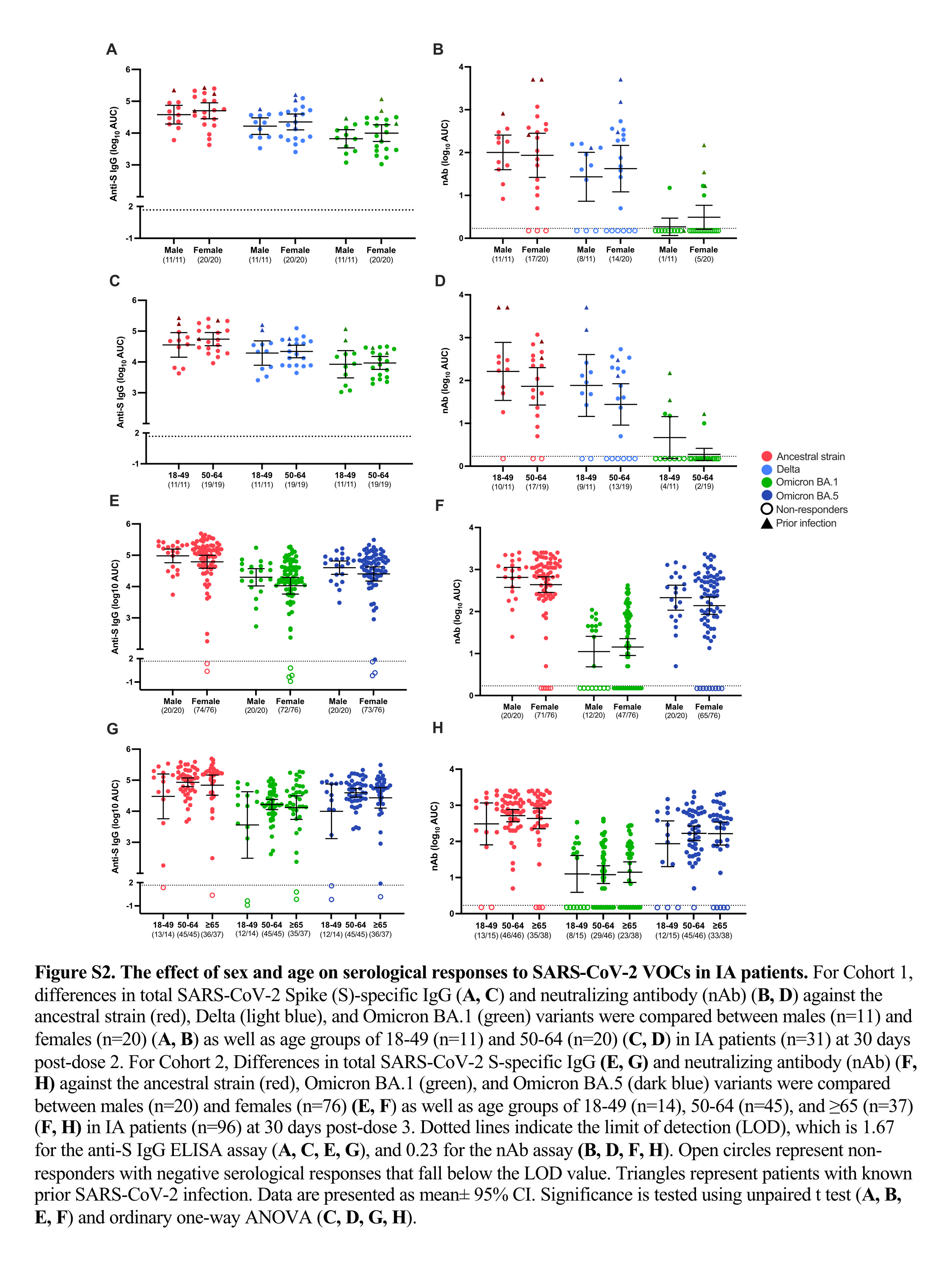

Supplement: Supplementary file 2 [file Image_2.tif]

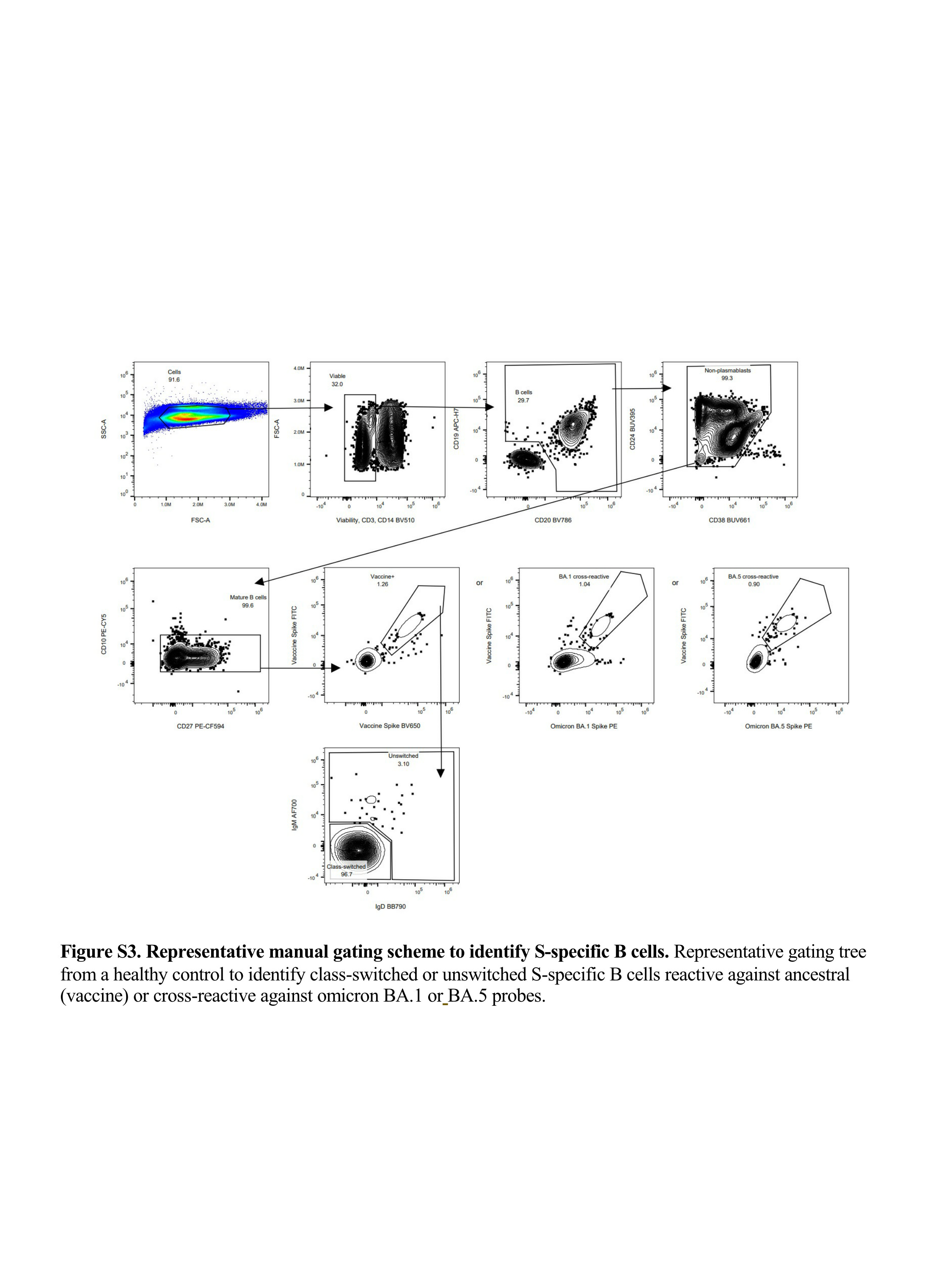

Supplement: Supplementary file 3 [file Image_3.tif]

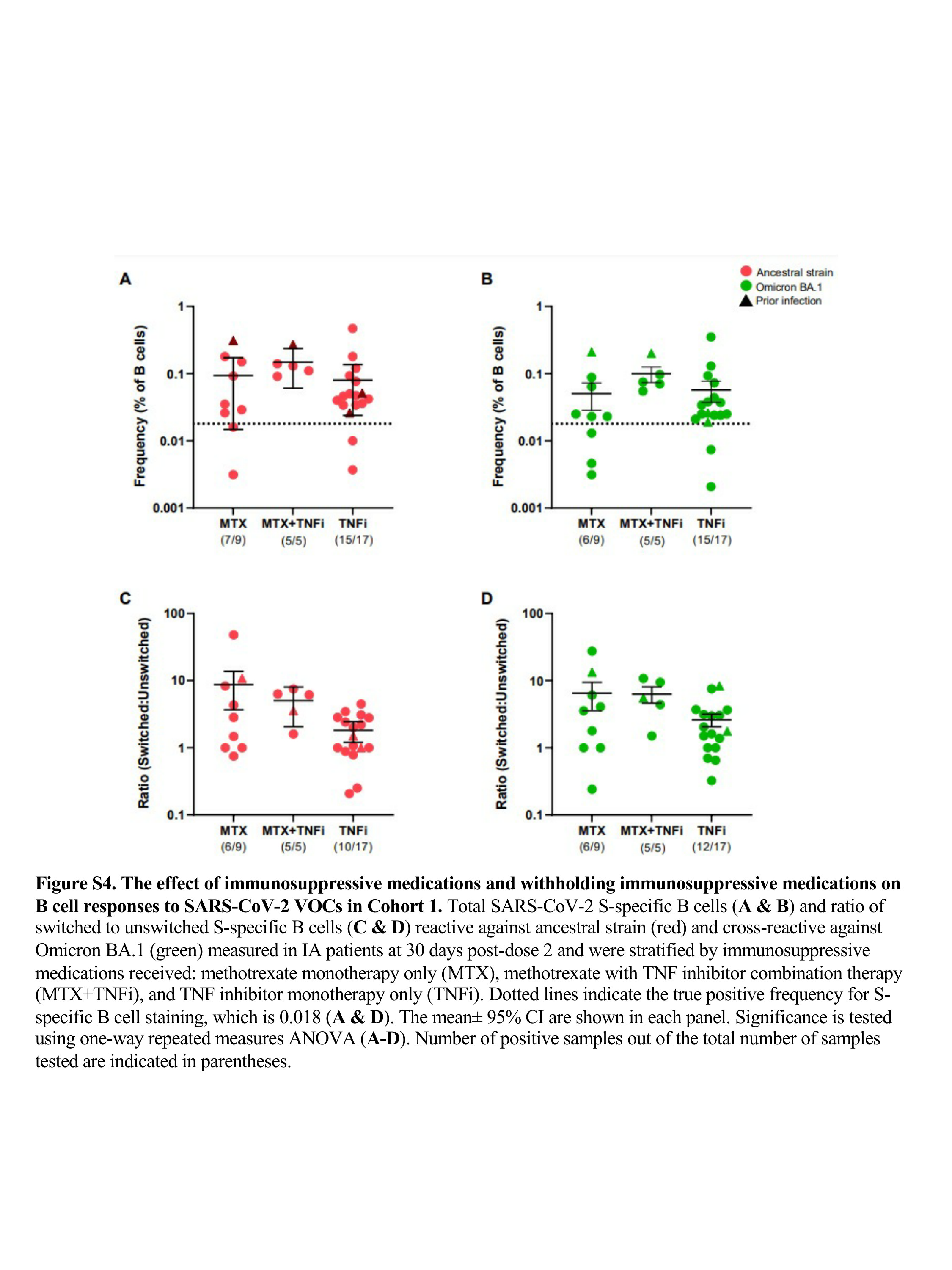

Supplement: Supplementary file 4 [file Image_4.tif]

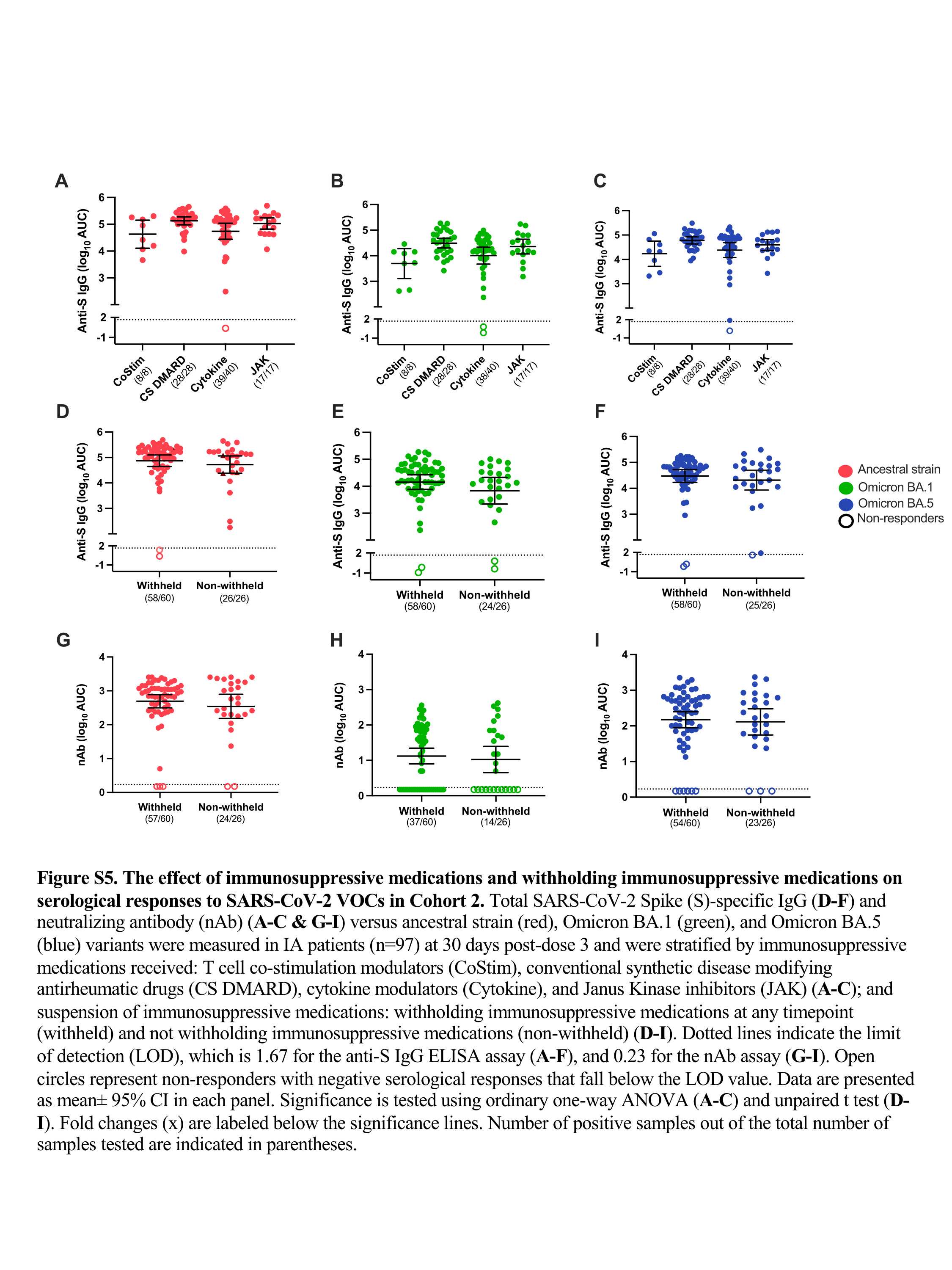

Supplement: Supplementary file 5 [file Image_5.tif]

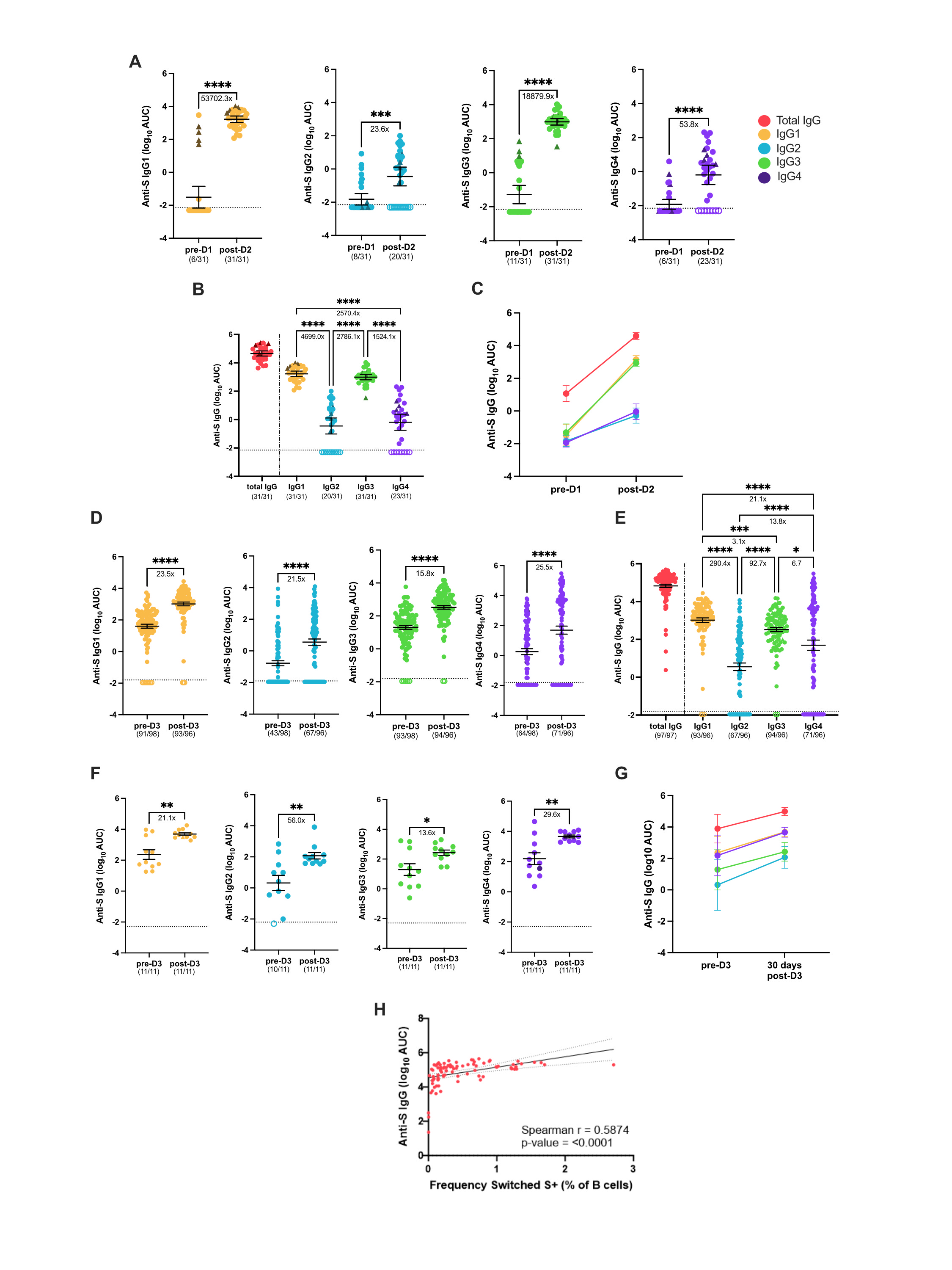

Supplement: Supplementary file 6 [file Image_6.tif]

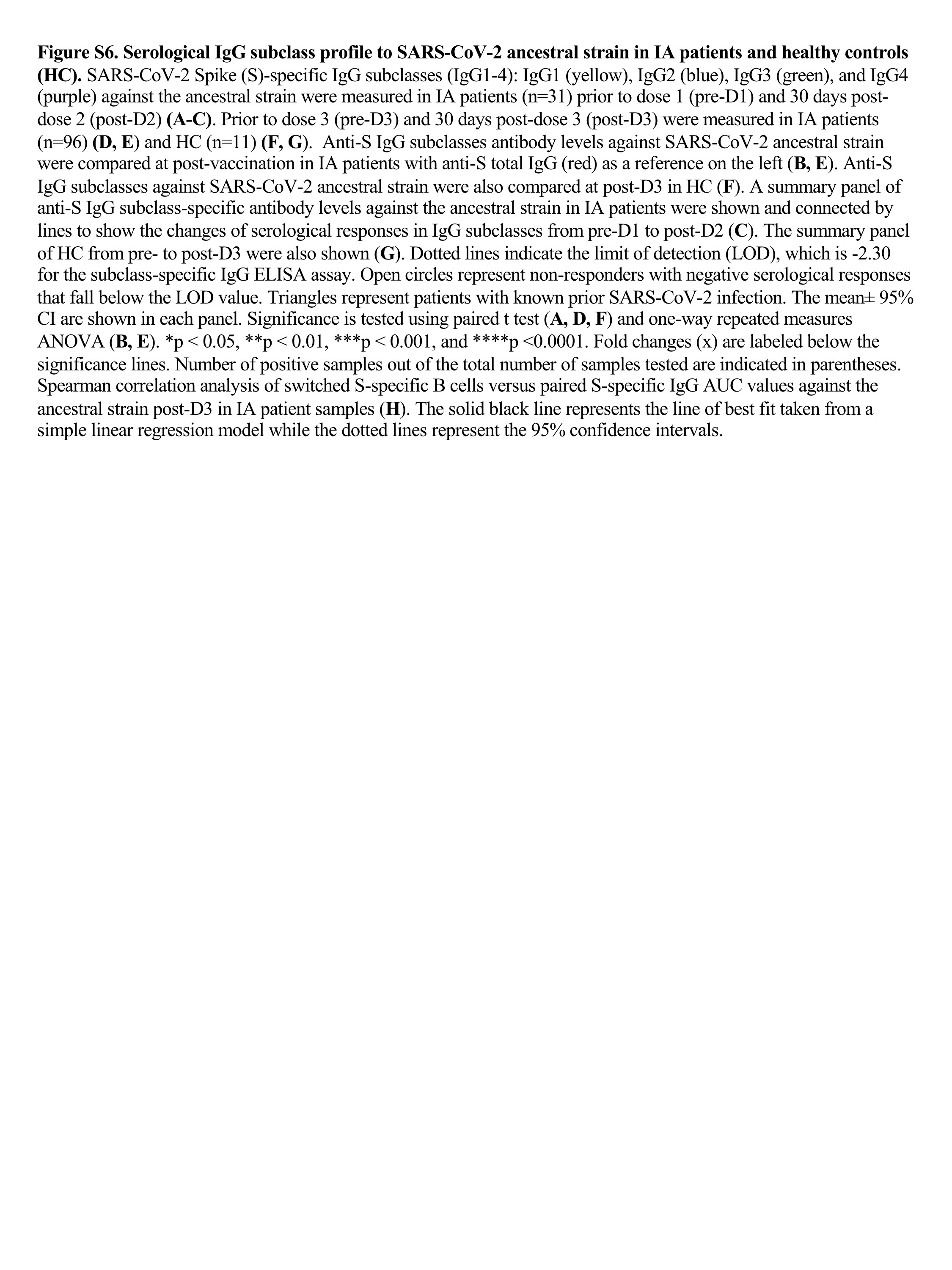

Supplement: Supplementary file 7 [file Image_7.tif]
